# Outcomes of adults hospitalized with COVID-19 at the University Teaching Hospital of Butare in Rwanda and validation of the Universal Vital Assessment (UVA) mortality risk score

**DOI:** 10.1371/journal.pgph.0003695

**Published:** 2024-12-09

**Authors:** Dona Fabiola Gashame, Kwame A. Akuamoah Boateng, Jean Damascene Twagirumukiza, Jean de Dieu Mahoro, Christopher C. Moore, Theogene Twagirumugabe

**Affiliations:** 1 Department of Anesthesia and Critical Care, Kigali University Teaching Hospital, University of Rwanda, Kigali, Rwanda; 2 Department of Surgery, Division of Acute Care Surgical Services, Virginia Commonwealth University School of Medicine, Richmond, Virginia, United States of America; 3 Research and Education Unit, Inkuru Nziza Orthopedic Specialized Hospital, Kigali, Rwanda; 4 Department of Anesthesia and Critical Care, University Teaching Hospital of Butare, University of Rwanda, Huye, Rwanda; 5 Department of Medicine, Division of Infectious Diseases and International Health, University of Virginia School of Medicine, Charlottesville, Virginia, United States of America; University of Global Health Equity, RWANDA

## Abstract

There are few data regarding clinical outcomes from COVD-19 from low-income countries (LICs) including Rwanda. Accordingly, we aimed to determine 1) outcomes of patients admitted to hospital with COVID-19 in Rwanda, and 2) the ability of the Universal Vital Assessment (UVA) score to predict mortality in patients with COVID-19 compared to sequential organ failure assessment (SOFA) and quick (qSOFA) scores. We conducted a retrospective study of patients aged ≥18 years hospitalized with laboratory-confirmed COVID-19 at the University Teaching Hospital of Butare (CHUB), Rwanda, April 2021-January 2022. For each participant, we calculated UVA, SOFA, and qSOFA risk scores and determined their area under the receive operating characteristic curve (AUC). We used logistic regression to determine predictors of mortality. Of the 150 patients included, 83 (55%) were female and the median (IQR) age was 61 (43–73) years. The median (IQR) length of hospital stay was 6 (3–10) days. Respiratory failure occurred in 69 (46%) including 34 (23%) who had ARDS. The case fatality rate was 44%. Factors independently associated with mortality included acute kidney injury (adjusted odds ratio [aOR] 7.99, 95% confidence interval [CI] 1.47–43.22, p = 0.016), severe COVID-19 (aOR 3.42, 95% CI 1.06–11.01, p = 0.039), and a UVA score >4 (aOR 7.15, 95% CI 1.56–32.79, p = 0.011). The AUCs for UVA, qSOFA, and SOFA scores were 0.86 (95% CI 0.79–0.92), 0.81 (95% CI 0.74–0.88), and 0.84 (95% CI 0.78–0.91), respectively, which were not statistically significantly different from each other. At a UVA score cut-off of 4, the sensitivity, specificity, positive predictive value, and negative predictive value for mortality were 0.58, 0.93, 0.86, and 0.74, respectively. Patients hospitalized with COVID-19 in CHUB had high mortality, which was accurately predicted by the UVA score. Calculation of the UVA score in patients with COVID-19 in LICs may assist clinicians with triage and other management decisions.

## Introduction

Coronavirus disease 2019 (COVID-19) caused by infection with severe acute respiratory syndrome coronavirus 2 (SARS-CoV-2) ranges from asymptomatic infection or mild upper respiratory symptoms to acute respiratory distress syndrome (ARDS), multi-organ dysfunction, and death [1]. In non-immune populations, the prevalence of respiratory failure among COVID-19 patients is approximately 20%, with 25% of these patients requiring admission to an intensive care unit (ICU) [2]. The risk factors for progression to severe COVID-19 are well described in high-income countries, but similar data from sub-Saharan Africa, and particularly from Rwanda, are limited [3,4].

The case fatality rate for severe COVID-19 is high, particularly among elderly patients and those with medical comorbidities [5]. Early detection and medical management are crucial to decrease mortality. Therefore, it is important to identify high-risk patients at admission. This process has been challenging in Rwanda due to limited medical and human resources [6]. Similar to other countries, during the initial stages of the pandemic, all patients with COVID-19 in Rwanda were admitted to hospital regardless of the severity of their illness. With a significant rise in cases, only severe cases, as well as those with medical comorbidities likely to predispose to severe COVID-19, were admitted to a dedicated high dependency unit (HDU) or ICU. The University Teaching Hospital of Butare (Centre Hospitalier Universitaire de Butare [CHUB]) was designated as a center for the care of severe COVID-19 cases in the southern and western regions of Rwanda.

The sequential organ failure assessment (SOFA) score, which relies on laboratory and clinical variables, has been used in highly-resourced settings to determine patient severity of illness in the ICU [7]. The simpler to calculate quick SOFA (qSOFA) score was derived for use outside the ICU and has been validated for use in low-income settings [8]. However, qSOFA is not recommended as a screening tool for sepsis due to lack of sensitivity and specificity [9]. To better understand mortality risk for hospitalized patients in sub-Saharan Africa (sSA), the Universal Vital Assessment (UVA) score was derived using data from patients in hospital-based cohort studies conducted in six African countries [10]. Its performance using easily obtainable clinical variables has since been validated throughout Africa including in Rwanda, where it has outperformed the qSOFA score [11]. To our knowledge, the UVA score has not been validated in patients with COVID-19; however, such a resource-appropriate tool to risk-stratify patients with COVID-19 in low-income countries (LICs) would be clinically useful and easy to implement. Accordingly, in this study, we aimed to determine 1) outcomes of patients admitted with COVID-19 to CHUB, and 2) the ability of the UVA score to predict their in-hospital mortality compared to qSOFA and SOFA scores.

## Methods

In this retrospective cross-sectional study, we collected data from patients with SARS-CoV-2 infection confirmed by a positive polymerase chain reaction or antigen rapid diagnostic test result obtained at or prior to admission admitted to the HDU or ICU at CHUB from April 20, 2021 through January 17, 2022. We excluded patients from the study who did not have oxygen saturation (SpO_2_) recorded within the first 48 hours of admission to the emergency ward, and we did not collect any further data from these patients (n = 33). The criteria for severe COVID-19 included ≥1 of the following: 1) SpO_2_ <94% while breathing ambient air, 2) ratio of arterial partial pressure of oxygen to fraction of inspired oxygen (PaO_2_/FiO_2_) <300 mm Hg, or SpO_2_/FiO_2_ ≤315 mmHg, 3) a respiratory rate >30 cycles per minute, or 4) lung infiltrates in >50% of the X-ray surface [12]. We defined respiratory failure as persistent hypoxemia with SpO_2_ <90% (PaO2 ≤ 50–60 mm Hg) despite receiving supplemental oxygen therapy at a rate of 10–15 L/minute. We defined acute kidney injury (AKI) as any of the following: an increase in serum creatinine by ≥0.3 mg/dl (≥26.5 μmol/l) within 48 hours; or an increase in serum creatinine to ≥1.5 times baseline, which was known or presumed to have occurred within the prior 7 days; or urine volume <0.5 ml/kg/h for 6 hours [13].

### Data collection

We collected demographic and clinical information, and extracted vital signs, laboratory results, and medical imaging findings obtained within 48 hours of admission. We calculated the SOFA score using the Kigali modification that includes SpO_2_/FiO_2_ <315, qSOFA, and UVA scores for each patient (S1 Table). We imputed 0 for missing values in each score. We grouped participants into predefined low, medium, and high-risk categories based on their UVA score (<2 = low risk, 2–4 = medium risk, and >4 = high risk) [10]. We documented pharmacological therapy received, respiratory support interventions (high-flow oxygen therapy, invasive mechanical ventilation, and noninvasive mechanical ventilation), renal replacement therapy, nutrition support, and patient outcomes, including the hospital length of stay, and in-hospital mortality.

### Statistical analysis

We did not conduct a formal sample size or power analysis as our exploratory retrospective analyses were based on a convenience sample of patients admitted to hospital with COVID-19. For data entry, we used Epidata version 3.1, and for analysis, we used SPSS version 25 and Python version 3.12.4. We reported categorical variables using frequency and percentage, and reported continuous variables as median with interquartile range (IQR). We assessed normality of continuous variables using the Kolmogorov-Smirnov and Shapiro-Wilk tests, both of which yielded p-values <0.001 for the outcome of interest confirming that the data were not normally distributed. Accordingly, we used the chi-square test to compare categorical variables, and the Mann Whitney U test to determine differences in median values for continuous variables. To determine discriminative performance for in-hospital mortality, we calculated the area under the receiver operating characteristic curve (AUC) for each clinical risk score and compared the results using the DeLong test. In our multivariable logistic regression analysis, we included variables with p<0.25 from the chi-square test, while adjusting for age, and qSOFA and UVA scores, and created calibration plots for each score. As a sensitivity analysis, we also created calibration plots from platt scaling, isotonic regression, and random forest analysis. We set statistical significance at p<0.05.

### Ethical considerations

The CHUB Institutional Research Board and College of Medicine and Health Sciences Research Ethics Committee approved the study with a waiver of consent (#REC/UTHB/088/2022). We accessed data for research purposes from April 4 through 15, 2022. DFG and TT had access to information that could identify individual participants during data collection, but this information was not stored and was not accessible during data analysis. Only deidentified data were used in the analysis.

## Results

**Fig 1:**
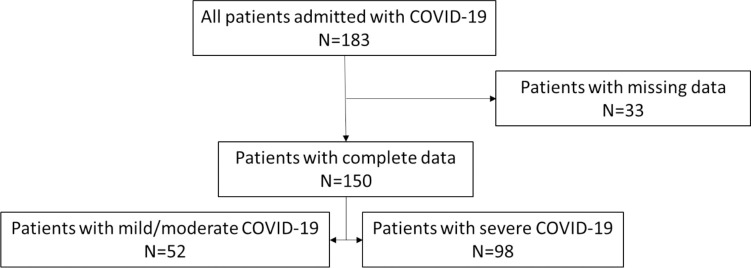
Flow diagram of patients included in analysis of outcomes of patients with COVID-19 at the time of admission to CHUB, May through October, 2021.

Of the 150 patients included in the study, 52 (35%) had mild or moderate COVID-19 and 98 (65%) had severe COVID-19 (Fig 1). The median (IQR) age was 61 (43–73) years. Patients with severe COVID-19 had a median (IQR) age of 64 (51–75) years compared to 50 (38–69) years for patients with mild or moderate COVID-19 (p = 0.008). Hypertension and diabetes mellitus were similarly prevalent among patients with severe COVID-19 and those with mild or moderate COVID-19 (Table 1). Patients with severe COVID-19 had a median (IQR) delay of 169 (96–192) hours in seeking medical care compared to a delay of 108 (48–192) hours in patients with mild to moderate COVID-19 (p = 0.08).

**Table 1:** Demographic and clinical characteristics of study participants with COVID-19 at the time of admission to CHUB, May through October, 2021.

	Missingness N (%)	Mild to moderate COVID-19* (N = 52)	Severe COVID-19* (N = 98)
**Demographics**			
Age (years)	0 (0)	50 (38–69)	64 (51–75)
Female	0 (0)	27 (52)	56 (57)
Time from symptoms to admission (hours)	19 (13)	108 (48–168)	169 (96–192)
**Comorbidities**			
Living with HIV	0 (0)	1 (2)	4 (4)
Diagnosed with tuberculosis	0 (0)	0 (0)	1 (1)
Hypertension	0 (0)	12 (23)	24 (24)
Diabetes mellitus	0 (0)	7 (13)	8 (8)
Liver disease	0 (0)	1 (2)	3 (3)
**Respiratory symptoms**			
Cough	0 (0)	29 (56)	71 (72)
Shortness of breath	0 (0)	17 (33)	65 (66)
Chest pain	0 (0)	17 (33)	65 (66)
Hemoptysis	0 (0)	0 (0)	4 (4)
Sore throat	0 (0)	0 (0)	1 (1)
**Gastrointestinal symptoms**			
Vomiting	0 (0)	5 (10)	6 (6)
Abdominal pain	0 (0)	7 (13)	5 (5)
Diarrhea	0 (0)	3 (6)	3 (3)
Nausea	0 (0)	3 (6)	3 (3)
Anorexia	0 (0)	9 (17)	3 (3)
**Central nervous system symptoms**			
Altered mental status	0 (0)	2 (4)	18 (18)
Headache	0 (0)	12 (23)	12 (12)
Seizures	0 (0)	1 (2)	5 (5)
Agitation	0 (0)	1 (2)	3 (3)
Dizziness	0 (0)	1 (2)	2 (2)
**Other symptoms**			
Weakness	0 (0)	27 (52)	33 (34)
Rhinorrhea	0 (0)	1 (2)	9 (9)
Chills	0 (0)	0 (0)	2 (2)
**Vital signs**			
Temperature (°C)	22 (15)	37.2 (36.2–38.8)	37.6 (36.6–38.7)
Respiratory rate ≥30 cycles/minute	0 (0)	0 (0)	35 (36)
Heart rate ≥120 beats/minute	0 (0)	3 (6)	21 (21)
Systolic blood pressure <90 mmHg	0 (0)	3 (6)	10 (10)
Glasgow coma scale score <15	0 (0)	6 (12)	42 (43)
**Laboratory values**			
Lymphocytes (10^9^/L)	16 (11)	1.2 (0.9–1.94)	0.91 (.62–1.60)
Hemoglobin (g/dL)	15 (10)	13.0 (12–15)	13.5 (11.8–15.2)
Creatinine (mmol/dl)	17 (13)	70.2 (60.8–87.6)	77.8 (60.1–119.1)
Sodium (mEq/L)	27 (18)	137 (134–139)	138 (132–142)
Alanine transaminase (IU/L)	27 (18)	27 (14–43)	39 (19–71)
**Risk scores**			
SOFA score	0 (0)	1 (0–2)	3 (3–6)
qSOFA <2	0 (0)	49 (94)	57 (58)
qSOFA ≥2	0 (0)	3 (7)	41 (93)
UVA score	0 (0)	0 (0–0)	3.5 (2–6)
UVA score <2	0 (0)	41 (79)	15 (15)
UVA score 2–4	0 (0)	10 (19)	40 (41)
UVA score >4	0 (0)	1 (2)	43 (44)

*Values are provided as n (%) or median (interquartile range).

Shortness of breath, chest pain, and altered mentation were more common in patients with severe COVID-19 (Table 1). The median (IQR) UVA score was 3.5 (2–6) among severe cases compared to 0 (0–0) among mild to moderate cases (p<0.001). The median (IQR) SOFA score was 3 (3–6) among severe cases compared to 1 (0–2) among mild and moderate cases (p<0.001). Dexamethasone was administered to all patients. There were 87 (58%) patients who received favipiravir and 8 (5%) who received tocilizumab. Proning was not considered as part of the standard of COVID-19 care and was not routinely performed or recorded at CHUB. Of the 98 patients with severe COVID-19, 61 (62%) developed respiratory failure, 34 (34%) developed ARDS and 9 (9%) developed septic shock. The median hospital stay was 6 days for both groups. Death occurred in 66 (44%) participants. The case fatality rate in patients with severe COVID-19 was 61% compared to 12% in patients with mild to moderate COVID-19 (odds ratio [OR] 12.10, 95% confidence interval [CI] 4.71–31.07, p<0.001) (Table 2).

**Table 2:** Association of clinical and socio-demographic characteristics with mortality among patients with COVID-19 admitted to hospital at CHUB, May through October, 2021.

	Survived n = 84	Died n = 66	Odds ratio	95% confidence interval	P value	Adjusted odds ratio	Adjusted 95% confidence interval	P value
Age >65 years	27 (61)	34 (52)	2.72	0.91–8.12	0.071	—	—	—
Female	45 (54)	38 (58)	1.07	0.62–1.84	0.058	—	—	—
Hypertension	17 (20)	19 (29)	1.59	0.75–3.38	0.23	—	—	—
Diabetes	10 (12)	14 (21)	1.52	0.52–4.42	0.45	—	—	—
Living with HIV	2 (2)	3 (4)	0.51	0.08–3.16	0.65	—	—	—
Tuberculosis	0 (0)	1 (1)	0.26	0.01–6.45	0.44	—	—	—
ARDS	8 (10)	22 (33)	2.55	1.16–5.59	0.020	—	—	—
Acute kidney injury	2 (2)	19 (29)	16.57	3.69–74.3	<0.001	7.99	1.47–43.22	0.016
Severe COVID-19	38 (45)	60 (91)	12.10	4.71–31.07	<0.001	3.42	1.06–11.01	0.039
qSOFA ≥2	7 (8)	37 (56)	11.0	4.34–27.8	<0.001	3.05	0.87–10.64	0.08
UVA <2	50 (60)	6 (9)	Reference		—	1.0	—	—
UVA 2–4	28 (33)	22 (33)	6.55	2.37–18.05	<0.001	2.79	0.87–8.95	0.085
UVA >4	6 (7)	38 (58)	32.5	15.77–176.6	<0.001	7.15	1.56–32.79	0.011

Of the 44 patients with qSOFA ≥2, 37 (84%) died compared to 29 (27%) of 106 patients with qSOFA <2 (OR 11.0, 95% CI 4.34–27.8, p<0.001). Of the 34 patients with ARDS, 21 (61%) died compared to 45 (38.7%) of 116 patients without ARDS (OR 2.55, 95% CI 1.16–5.59, p = 0.020). Of the 21 patients with AKI, 19 (90%) died compared to 2 (9%) patients without AKI (OR 16.57, 95% CI, 3.69–74.3, p<0.001). Of the 98 patients with severe COVID-19, 60 (61.22%) died compared to 38 (38.78%) patients who had mild to moderate COVID-19 (OR 12.10, 95% CI 4.71–31.07, p <0.001). There was no statistically significant difference in mortality according to age, sex, or presence of medical comorbidities (Table 2).

**Fig 2:**
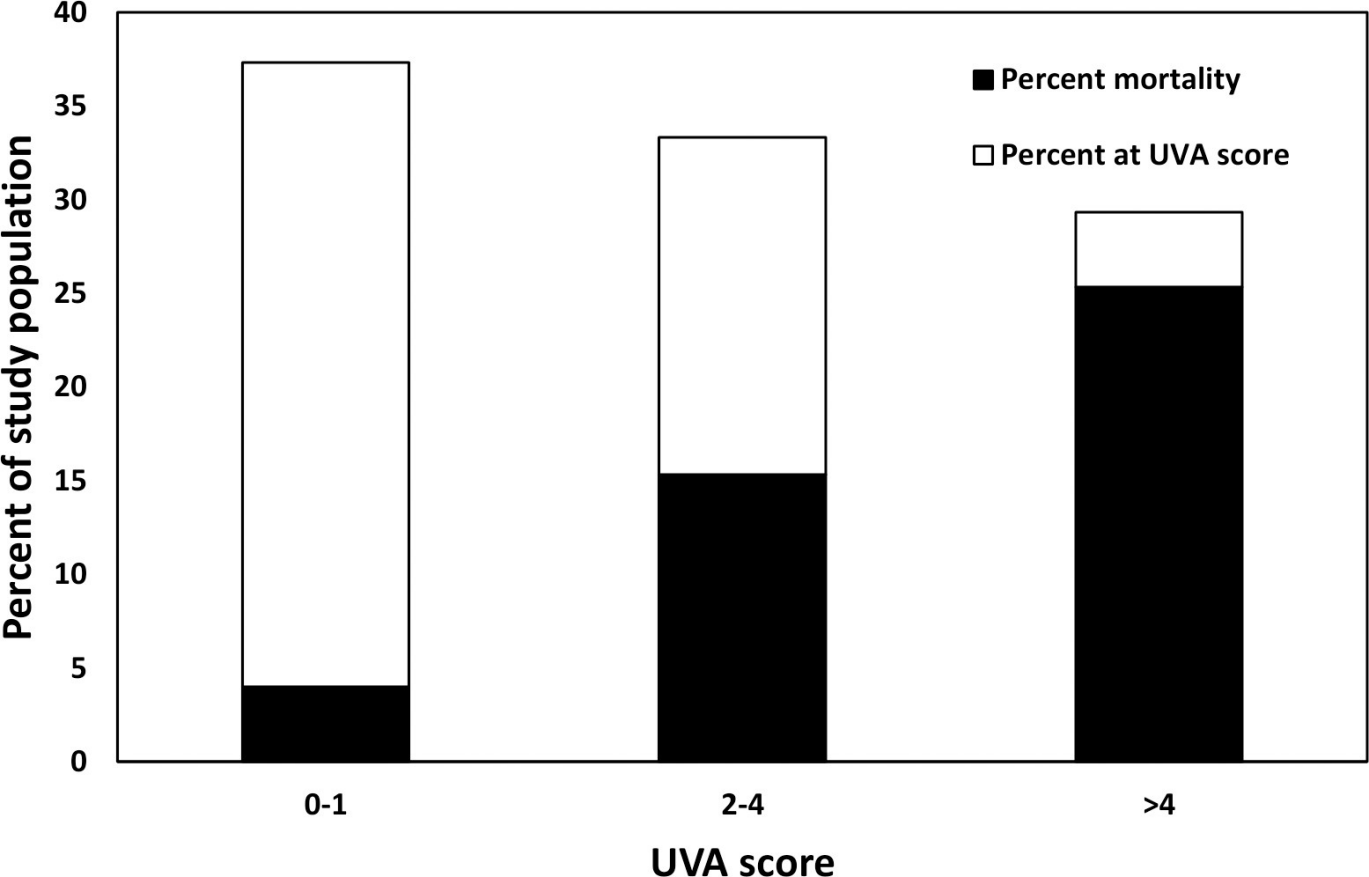
The frequency and associated case fatality rate of UVA score risk categories (low 0–1, medium 2–4, high >4) of patients with COVID-19 at the time of admission to CHUB, May through October, 2021.

**Fig 3:**
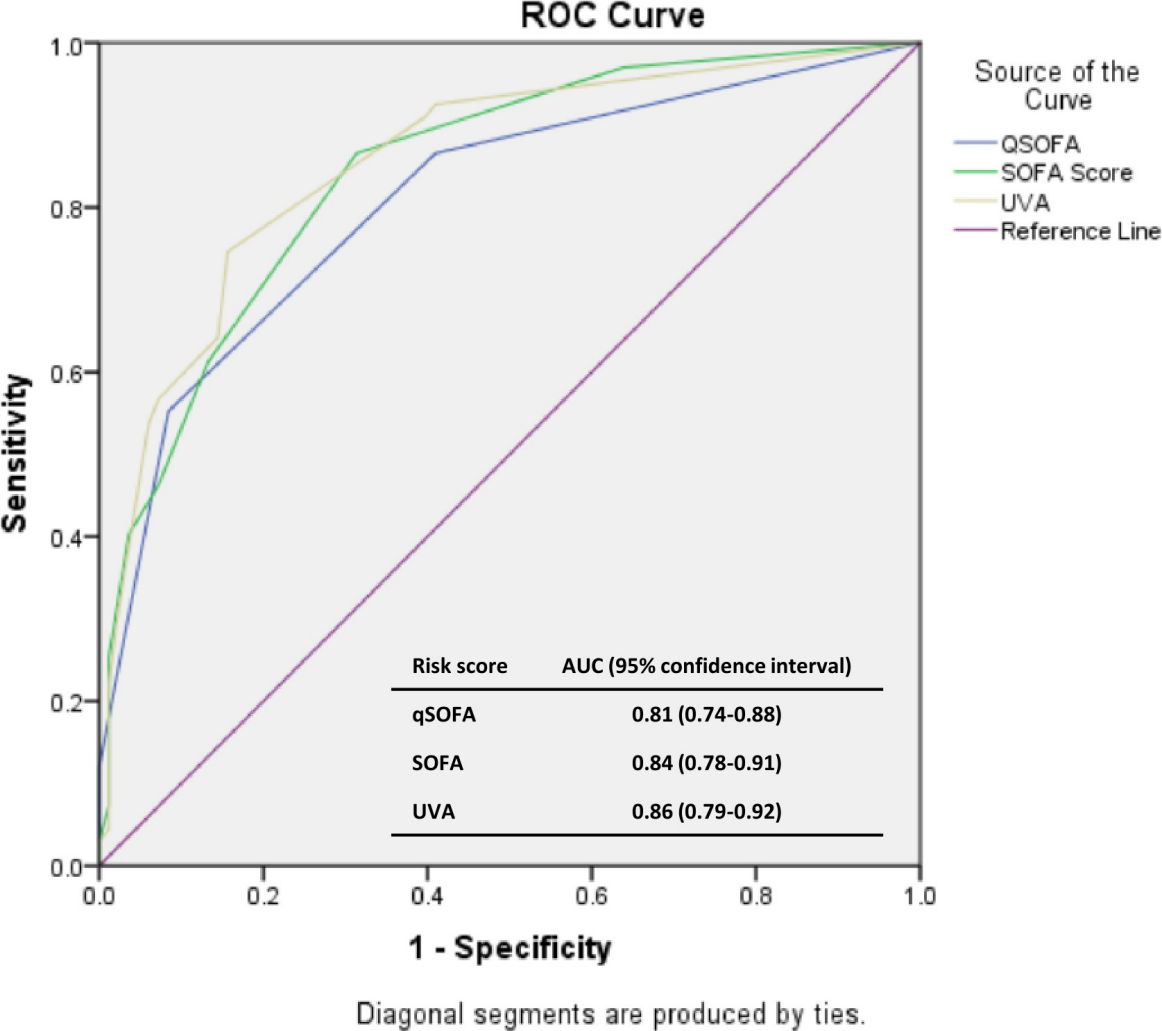
Receiver operating characteristic curves for qSOFA, SOFA, and UVA risk scores among patients with COVID-19 at the time of admission to CHUB, May through October, 2021.

Compared to patients with a UVA score <2, patients with a UVA score 2–4 (OR 5.172, 95% CI 1.12–22.33, p = 0.03) or a UVA score >4 (OR 16.85, 95% CI 3.01–94.23 p = 0.001) had a higher risk of death (Fig 2 and Table 3). The AUCs for UVA, qSOFA, and SOFA scores were 0.86 (95% CI 0.79–0.92), 0.81 (95% CI 0.74–0.88), and 0.84 (95% CI 0.78–0.91), respectively, which were not statistically significantly different from each other (Fig 3). At a UVA score cut-off of 4, the sensitivity, specificity, positive predictive value, and negative predictive value for mortality were 0.58, 0.93, 0.86, and 0.74, respectively. In the multivariable logistic regression analysis, a UVA score >4 (adjusted OR [aOR] 7.15, 95% CI 1.56–32.79, p = 0.011), AKI (aOR 7.99, 95% CI 1.47–43.22, p<0.001), and severe COVID-19 (aOR 3.42, 95% CI 1.06–11.01, p = 0.04) were independently associated with mortality (Table 2 and S1 Fig). We did not include ARDS in the multivariable model due to concern for collinearity with severe COVID-19.

**Table 3:** A multivariable analysis of the differences between the UVA cut-offs in their associations with mortality among patients with COVID-19 admitted to hospital at CHUB, May through October, 2021.

Comparator score cut-offs		Odds ratio	95% Confidence interval	P value
UVA 2–4	UVA 0–1	7.1	2.6–19.6	<0.0001
UVA >4	UVA 0–1	52.8	15.8–176.6	<0.0001
UVA >4	UVA 2–4	7.4	2.7–20.7	<0.0001

## Discussion

In this study, we found that hospitalized patients with COVID-19 had a high case fatality rate of 44%, which increased to 61% in patients admitted with severe COVID-19. Independent risk factors for mortality included AKI, severe COVID-19, and a UVA score >4. The UVA and qSOFA scores had good discrimination for mortality which was similar to that of SOFA and both UVA and qSOFA scores could be easily used to risk-stratify patients admitted to hospital with COVID-19 in Rwanda.

We found a non-statistically significant increase in age in patients with severe COVID-19 and those who died, which may be due to the admission of primarily elderly patients, regardless of the severity of their COVID-19 at the time of admission, in order to ensure close monitoring and prompt management if their clinical condition worsened. This finding aligns with previous cohort studies and a systematic review, which indicated that advanced age is associated with more severe symptoms and a higher rate of complications among COVID-19 patients [14,15]. Additionally, In a 2021 study from Uganda, age ≥50 years was associated with all-cause in-hospital mortality [16]. In the setting of alveolar damage, aging is linked to an increase in the expression of Angiotensin-Converting Enzyme-2 (ACE-2), the receptor that binds the SARS-CoV-2 spike protein [17]. Severe outcomes among the elderly may be explained by the direct and indirect pathways through which SARS-CoV-2 induces lung injury and limited physiological reserves. This process leads to increased inflammatory responses, cell death, and subsequent respiratory failure. Inhibiting ACE-2 may mitigate SARS-CoV-2 infection-induced pathological alterations in the lung that results in acute lung injury and severe pneumonia [18,19].

In our study, there was an increase in the prevalence of hypertension and diabetes in patients who died from COVID-19 compared to those who survived. This increase was not statistically significant, likely due to small numbers; however, comorbidities, including hypertension and diabetes mellitus, are often associated with severe COVID-19 [20]. In a study from Uganda in 2020, hypertension, diabetes, and elevated body mass index were commonly found in patients admitted to hospital with COVID-19 [21]. In the 2021 study from Uganda, the finding of any comorbidity was associated with in-hospital mortality [16]. A study conducted in Rwanda in 2021 found that severe COVID-19 status was associated with age and the number of patient comorbidities. Patients aged above 60 years and those between the age of 51 and 60 were respectively 12 and 7 times more likely to have severe COVID-19 compared to those aged below 30 years. Having two comorbidities doubled the risk of developing a severe COVID-19 status compared to those with no comorbidity [22]. In a multicenter, prospective, observational cohort study of patients with COVID-19 that included health facilities in 10 African countries, risk factors that were independently associated with mortality included increasing age, HIV/AIDS, diabetes, chronic liver disease, and kidney disease [4].

The 44% case fatality rate among all patients with COVID-19 and 61% among those with severe COVID-19 was high. Our findings were similar to the case fatality rate of 41% found in the multi-site prospective cohort study of patients with COVID-19 admitted to an HDU or ICU across Africa [4]. Shortness of breath and chest pain associated with respiratory failure were the most prevalent symptoms of severe COVID-19 in our study. In a meta-analysis of clinical characteristics of 4499 COVID-19 patients in Africa, common symptoms included fever (43%), cough (33%), breathing problems (17%), and headache (11%) [23]. Complications including AKI and ARDS were independently associated with mortality in our study and likely reflected a high severity of disease and possibly limited critical care resources to support organ failure through renal replacement and mechanical ventilation [4,24].

UVA, qSOFA, and SOFA scores were higher in patients with severe COVID-19 compared to those with mild or moderate COVID-19. The UVA score had good discrimination for mortality with an AUC of 0.85. While this is the first study to evaluate UVA in patients with COVID-19, other studies have found that the National Early Warning Score 2 (NEWS2) had an AUC of 0.71, 0.77, and 0.87 in patients in the United Kingdom, South Africa, and Italy, respectively [25–27]. Other clinical predictive tools, including SOFA and SAPS II, can be useful to assess organ failure during COVID-19, but require diagnostic testing which is often not available in LICs [28]. In this context, the UVA score, which is based on physiological changes without laboratory investigations and has been validated in Rwanda with good discrimination of mortality in hospitalized adults with and without COVID-19, could be useful for implementation in similar patient populations [11].

This study had limitations. First, this was a retrospective single center study that took place in Rwanda and our analyses were based on a convenience sample of patients admitted to hospital with COVID-19. This was due to the episodic nature of the epidemic. Data collection occurred during a specific period, and the SARS-CoV-2 variants have differed from one wave to another, leading to varying outcomes. Further validation of the UVA and other risk scores to predict mortality risk of COVID-19 should take place at other centers in sub-Saharan Africa. Second, the relatively small size of our patient cohort may limit the generalizability of our results. However, there are few studies of COVID-19 outcomes or mortality risk scores that have been evaluated in African populations, so this study provides new insight and a benchmark for future studies. Third, our patient cohort was likely relatively non-immune to SARS-CoV-2 and subsequent patients with COVID-19 are likely to have some immunity to SARS-CoV-2, either through vaccination or infection, which will decrease disease severity and improve outcomes. Nonetheless, the UVA score had good discrimination of mortality in patients with COVID-19.

Overall, we found a high case fatality rate in patients requiring hospitalization for COVID-19 in Rwanda. Mortality from COVID-19 was predicted by higher UVA score and associated with AKI and severe COVID-19 at admission to CHUB. The UVA score could be implemented to risk stratify patients with COVID-19 who are admitted to hospital in Rwanda.

## Supporting information

S1 Fig Calibration plots of UVA, qSOFA, and SOFA score models for the prediction of in-hospital mortality of patients with COVID-19 at the time of admission to CHUB, May through October, 2021. The dashed blue line indicates perfect calibration; the solid blue, orange, green, and red lines represent logistic regression, platt scaling, isotonic regression, and random forest models, respectively.
(TIFF)

S1 Table Universal Vital Assessment (UVA) and quick Sequential Organ Failure Assessment (qSOFA) score components, cut-offs, and associated points. (DOCX)

S1 Data (XLSX)
