# Supplementary figures and images for: Outcomes of adults hospitalized with COVID-19 at the University Teaching Hospital of Butare in Rwanda and validation of the Universal Vital Assessment (UVA) mortality risk score
Source: PLOS Glob Public Health. 2024 Dec 9;4(12):e0003695. doi: 10.1371/journal.pgph.0003695 (PMC11627434; doi:10.1371/journal.pgph.0003695)

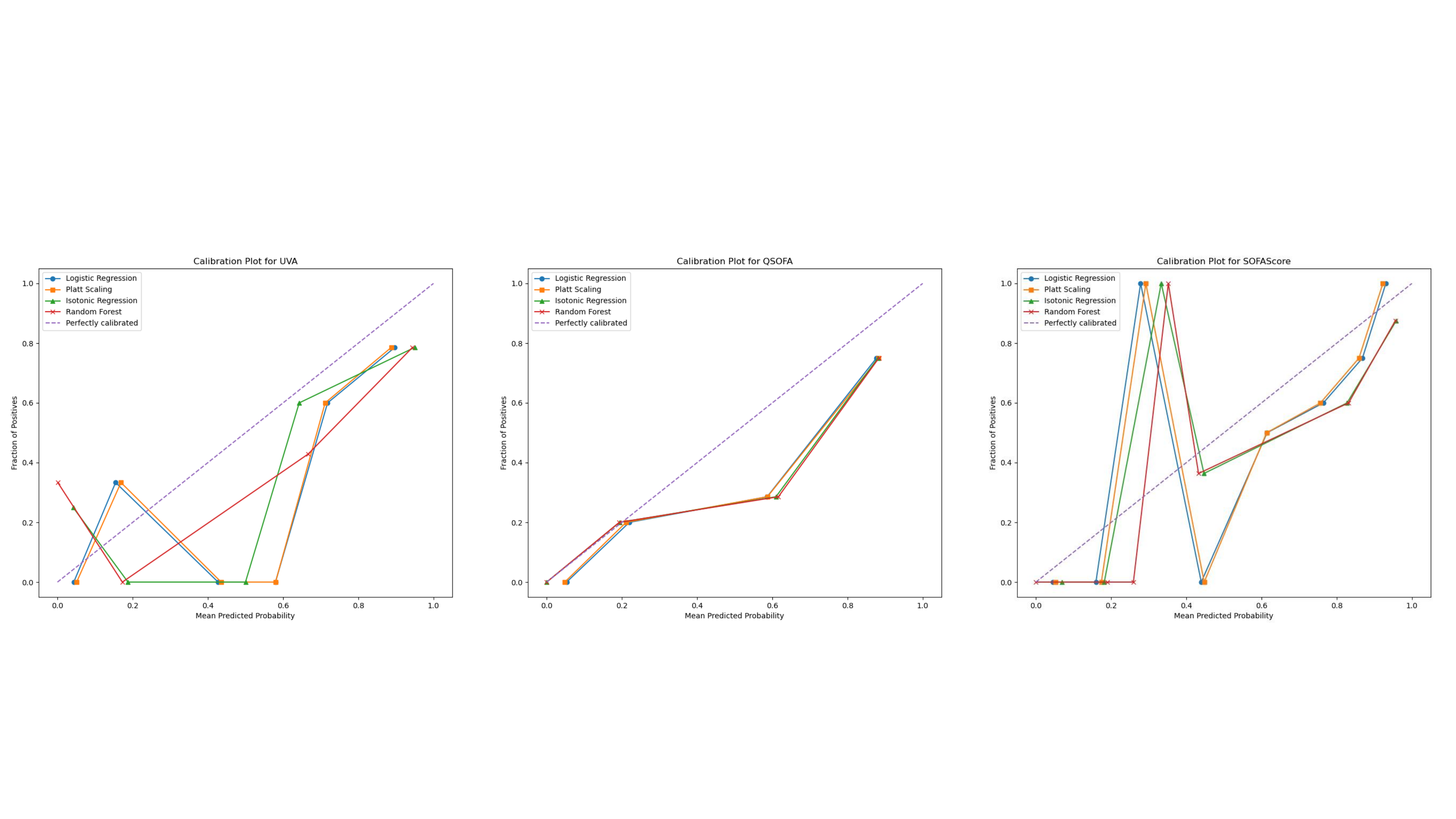

Supplement: S1 Fig — The dashed blue line indicates perfect calibration; the solid blue, orange, green, and red lines represent logistic regression, platt scaling, isotonic regression, and random forest models, respectively. (TIFF) [file pgph.0003695.s001.tiff]
