# Supplementary material for: Outcomes of adults hospitalized with COVID-19 at the University Teaching Hospital of Butare in Rwanda and validation of the Universal Vital Assessment (UVA) mortality risk score
Source: PLOS Glob Public Health. 2024 Dec 9;4(12):e0003695. doi: 10.1371/journal.pgph.0003695 (PMC11627434; doi:10.1371/journal.pgph.0003695)
Supplement: S1 Table — (DOCX) [file pgph.0003695.s002.docx]

**Supplementary Table 1.** Universal Vital Assessment (UVA) and quick Sequential Organ Failure Assessment (qSOFA) score components, cut-offs, and associated points.

| **Score component** | **UVA cut-off** | **Points** | **qSOFA cut-off** | **Points** |
| --- | --- | --- | --- | --- |
| Systolic blood pressure (mmHg) | <90 | 1 | ≤100 | 1 |
| Respiratory rate (brpm) | ≥30 | 1 | ≥22 | 1 |
| GCS (score) | <15 | 4 | <15 | 1 |
| Heart rate (bpm) | ≥120 | 1 | --- | --- |
| Temperature (^o^C) | <36 | 2 | --- | --- |
| Oxygen saturation | <92 | 2 | --- | --- |
| Living with HIV | Yes | 2 | --- | --- |
| Bpm, beats per minute; brpm, breaths per minute; GCS, Glasgow coma scale. | | | | |
